# Marital status and survival of patients with oral cavity squamous cell carcinoma: a population-based study

**DOI:** 10.18632/oncotarget.16095

**Published:** 2017-03-10

**Authors:** Xiao Shi, Ting-ting Zhang, Wei-ping Hu, Qing-hai Ji

**Affiliations:** ^1^ Department of Head and Neck Surgery, Fudan University Shanghai Cancer Center, Shanghai, China; ^2^ Department of Oncology, Shanghai Medical College, Fudan University, Shanghai, China; ^3^ Department of Respiratory Medicine, Zhongshan Hospital, Fudan University, Shanghai, China

**Keywords:** oral cavity squamous cell carcinoma, marital status, SEER, survival analysis, spousal support

## Abstract

**Background:**

The relationship between marital status and oral cavity squamous cell carcinoma (OCSCC) survival has not been explored. The objective of our study was to evaluate the impact of marital status on OCSCC survival and investigate the potential mechanisms.

**Results:**

Married patients had better 5-year cancer-specific survival (CSS) (66.7% vs 54.9%) and 5-year overall survival (OS) (56.0% vs 41.1%). In multivariate Cox regression models, unmarried patients also showed higher mortality risk for both CSS (Hazard Ratio [HR]: 1.260, 95% confidence interval (CI): 1.187–1.339, *P* < 0.001) and OS (HR: 1.328, 95% CI: 1.266–1.392, *P* < 0.001). Multivariate logistic regression showed married patients were more likely to be diagnosed at earlier stage (*P* < 0.001) and receive surgery (*P* < 0.001). Married patients still demonstrated better prognosis in the 1:1 matched group analysis (CSS: 62.9% vs 60.8%, OS: 52.3% vs 46.5%).

**Materials and Methods:**

11022 eligible OCSCC patients were identified from Surveillance, Epidemiology, and End Results (SEER) database, including 5902 married and 5120 unmarried individuals. Kaplan-Meier analysis, Log-rank test and Cox proportional hazards regression model were used to analyze survival and mortality risk. Influence of marital status on stage, age at diagnosis and selection of treatment was determined by binomial and multinomial logistic regression. Propensity score matching method was adopted to perform a 1:1 matched cohort.

**Conclusions:**

Marriage has an independently protective effect on OCSCC survival. Earlier diagnosis and more sufficient treatment are possible explanations. Besides, even after 1:1 matching, survival advantage of married group still exists, indicating that spousal support from other aspects may also play an important role.

## INTRODUCTION

Approximately over 200,000 new cases are diagnosed with oral cavity cancer (OCC) annually in the world and OCC leads to more than 100,000 deaths globally [1]. Oral cavity squamous cell carcinoma (OCSCC) accounts for the vast majority of total OCC and is generally considered to be closely related with tobacco and alcohol use [2]. In the United States, the incidence of OCSCC decreases slightly in recent years [3, 4], however in Australia and many regions of Western Europe, the morbidity continues to increase [5, 6]. It's also noteworthy that in developing countries, OCSCC is still one of the ten leading causes for cancer death in male population [1].

It is a common understanding that social support, especially spousal support plays an important role in improving cancer outcomes. Many researches in recent years have demonstrated that marital status could independently affect survival in several cancer types [7–11]. Similarly, Inverso G et al. reported married patients with head and neck cancer had a longer survival than those unmarried, however in that study, researchers only took cancer-specific survival (CSS) into consideration while neglected overall survival (OS), which might benefit more from spousal support. Additionally, they were not able to discuss if marriage would affect survival from other aspects besides of favoring early diagnosis and adequate treatment [12]. Therefore, to the best of our knowledge, there is a dearth of researches focusing on the significance of marital status on OCSCC survival and elucidating relatively comprehensive mechanisms. In this study, we used data from Surveillance, Epidemiology, and End Results (SEER) database to investigate the influence of marital status on OCSCC survival and propose potential explanations in detail.

## RESULTS

### Patient baseline characteristics

Totally, we identified 11022 eligible OCSCC patients diagnosed between 1988 and 2008 in our study. All the patients in our study were actively followed up. Of these 11022 cases, 5902 (53.5%) were married and 5120 (46.5%) were unmarried. 2.7% of unmarried patients came up with distant metastasis while the metastatic rate in the married group was 1.5%. Demographics and clinicopathological characteristics of enrolled patients are listed in Table 1. Significant differences (*P* < 0.001) were observed in all variables by Pearson Chi-squared (χ^2^) test except for year (*P* = 0.098) and grade (*P* = 0.138). For better analysis, we listed stage IVc which stands for distant metastasis (M1) apart from Stage IVa/IVb because of different prognosis. We observed that patients in the married group were more likely to be white and male and had a higher proportion of tongue cancer. Besides, in general, married group also displayed earlier stage at diagnosis and a higher rate of receiving surgery, however, unmarried patients seemed more likely to be treated with radiotherapy than those married (Table 1).

**Table 1:** Baseline characteristics of OCSCC patients by marital status

Characteristic	Total	Married	Unmarried	*P*
	11022 (100)	5902 (100)	5120 (100)	
**Gender**				< 0.001
Female	4163 (37.8)	1909 (32.3)	2254 (44.0)	
Male	6859 (62.2)	3993 (67.7)	2866 (56.0)	
**Age**				< 0.001
< 35	226 (2.1)	107 (1.8)	119 (2.3)	
35–44	764 (6.9)	412 (7.0)	352 (6.9)	
45–54	2164 (19.6)	1156 (19.6)	1008 (19.7)	
55–64	2909 (26.4)	1647 (27.9)	1262 (24.6)	
65–74	2587 (23.5)	1532 (26.0)	1055 (20.6)	
75–84	1785 (16.2)	860 (14.6)	925 (18.1)	
> 85	587 (5.3)	188 (3.2)	399 (7.8)	
**ICD-O-3 site code^1^**				< 0.001
Tongue^2^	3963 (36.0)	2293 (38.9)	1670 (32.6)	
Gum and other mouth	3846 (34.9)	2035 (34.5)	1811 (35.4)	
Floor of mouth	3213 (29.2)	1574 (26.7)	1639 (32.0)	
**Race**				< 0.001
White	9041 (82.0)	4944 (83.8)	4097 (80.0)	
Black	1089 (9.9)	351 (5.9)	738 (14.4)	
Other^3^	856 (7.8)	589 (10.0)	267 (5.2)	
Unknown	36 (0.3)	18 (0.3)	18 (0.4)	
**Grade**				0.138
Well/Moderately differentiated	8681 (78.8)	4679 (79.3)	4002 (78.2)	
Poorly/Undifferentiated	1906 (17.3)	1011 (17.1)	895 (17.5)	
Unknown	435 (3.9)	212 (3.6)	223 (4.4)	
**Year**				0.098
1988–1994	1540 (14.0)	857 (14.5)	683 (13.3)	
1995–2001	3172 (28.8)	1660 (28.1)	1512 (29.5)	
2002–2008	6310 (57.2)	3385 (57.4)	2925 (57.1)	
**TNM Stage**				< 0.001
I	3591 (32.6)	2200 (37.3)	1391 (27.2)	
II	2201 (20.0)	1162 (19.7)	1039 (20.3)	
III	1263 (11.5)	613 (10.4)	650 (12.7)	
IVa/IVb	3741 (33.9)	1837 (31.1)	1904 (37.2)	
IVc	226 (2.1)	90 (1.5)	136 (2.7)	
**Surgery**				< 0.001
No surgery	1972 (17.9)	797 (13.5)	1175 (22.9)	
Local excision/destruction^4^	1953 (17.7)	1131 (19.2)	822 (16.1)	
Wide/Radical excision	7097 (64.4)	3974 (67.3)	3123 (61.0)	
**Radiotherapy**				< 0.001
No	5490 (49.8)	3087 (52.3)	2403 (46.9)	
Yes	5532 (50.2)	2815 (47.7)	2717 (53.1)	

SEER 1988–2008 (*n* = 11022).

^1^Lip was not included because NCCN regards lip and oral cavity as two different parts and provides different treatment guidelines respectively.

^2^Subsites of the tongue regarded as anatomic part of oropharynx were not included.

^3^Includes American Indian/Alaska Native, Asian/Pacific Islander.

^4^Local destruction includes photodynamic therapy, electrocautery, cryosurgery, laser ablation, etc.

### Impact of marital status on cancer-specific survival of OCSCC patients

**Figure 1:**
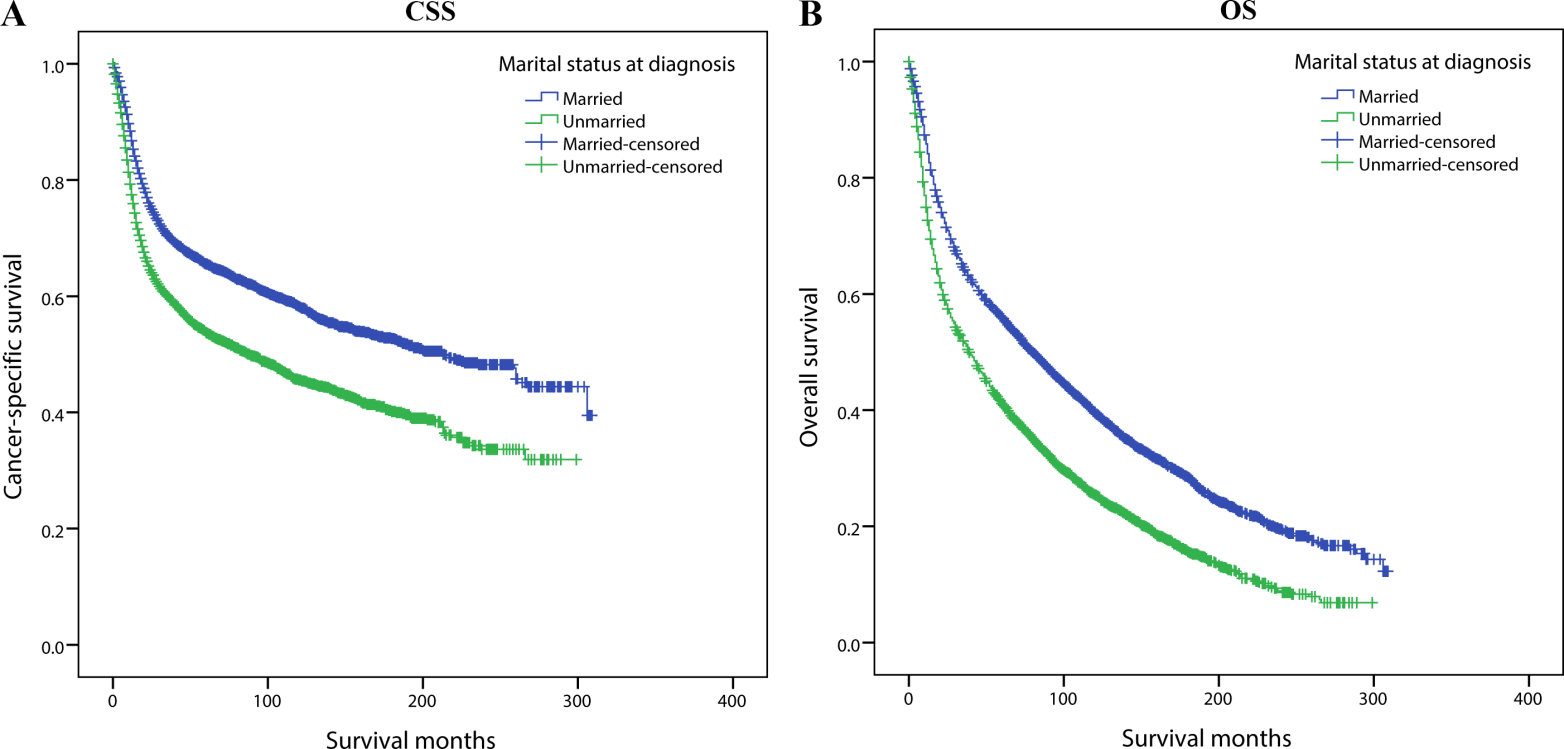
Kaplan-Meier survival curves:cancer-specific survival and overall survival in 11022 OCSCC patients Married vs Unmarried (**A**) Cancer-specific survival: χ^2^ = 235.220, *P* < 0.001; (**B**) Overall survival: χ^2^ = 329.475, *P* < 0.001.

We used Kaplan-Meier analysis and Log-rank test to evaluate the impact of marital status on CSS of OCSCC patients (Figure 1A). In summary, the married group had a better 5-year CSS (66.7% vs 54.9%) than those unmarried. These prognostic differences were also significant in the univariate Log-rank test (*P* < 0.001). In the univariate analysis, gender (*P* = 0.013), age (*P* < 0.001), site (*P* < 0.001), race (*P* < 0.001), grade (*P* < 0.001), TNM stage (*P* < 0.001), surgery (*P* < 0.001) and radiotherapy (*P* < 0.001) were also significantly associated with cancer-specific survival of OCSCC patients and these variables were all included in the following multivariate Cox analysis (Table 2).

**Table 2:** Univariate and multivariate analysis for evaluating the CSS predictors of OCSCC patients

Variable	5-year CSS	Univariate Analysis		Multivariate Analysis		
		Log-rank χ^2^	*P*	HR	95% CI	*P*
**Marital status**		183.135	< 0.001			
Married	66.7%			Reference		
Unmarried	54.9%			1.26	1.187–1.339	< 0.001
**Gender**		6.189	0.013			
Female	62.1%			Reference		
Male	59.7%			1.068	1.003–1.138	0.041
**Age**		227.992	< 0.001			
< 35	74.7%			0.326	0.246–0.432	< 0.001
35–44	66.9%			0.47	0.396–0.558	< 0.001
45–54	64.7%			0.497	0.431–0.573	< 0.001
55–64	62.9%			0.543	0.474–0.622	< 0.001
65–74	61.7%			0.612	0.534–0.700	< 0.001
75–84	53.8%			0.815	0.710–0.934	0.003
> 85	45.6%			Reference		
**ICD-O-3 site code**		109.635	< 0.001			
Tongue	66.7%			Reference		
Gum and other mouth	57.6%			0.964	0.896–1.037	0.326
Floor of mouth	58.9%			1.096	1.016–1.182	0.017
**Race**		139.129	< 0.001			
White	63.2%			Reference		
Black	46.8%			1.228	1.123–1.343	< 0.001
Other	58.9%			1.134	1.018–1.263	0.022
Unknown	82.7%			0.252	0.081–0.781	0.017
**Grade**		190.713	< 0.001			
Well/Moderately differentiated	64.3%			Reference		
Poorly/Undifferentiated	47.6%			1.351	1.259–1.451	< 0.001
Unknown	60.5%			0.945	0.807–1.106	0.481
**Year**		2.019	0.364			NI
1988–1994	60.5%					
1995–2001	61.9%					
2002–2008	61.2%					
**TNM Stage**		1529.025	< 0.001			
I	81.5%			Reference		
II	65.8%			1.477	1.339–1.629	< 0.001
III	52.8%			1.974	1.769–2.204	< 0.001
IVa/IVb	43.7%			2.583	2.370–2.814	< 0.001
IVc	18.2%			4.871	4.111–5.771	< 0.001
**Surgery**		1392.293	< 0.001			
No surgery	30.2%			Reference		
Local excision/destruction	74.7%			0.458	0.413–0.509	< 0.001
Wide/Radical excision	65.5%			0.499	0.464–0.536	< 0.001
**Radiotherapy**		557.125	< 0.001			
No	68.3%			Reference		
Yes	58.2%			0.716	0.669–0.767	< 0.001

SEER 1988–2008 (*n* = 11022).

Abbreviations: HR: Hazard ratio; CI: confidence interval; NI: Not Included.

In the Cox regression, we found that unmarried group had a significantly increasing risk for cancer-specific mortality (HR 1.260, 95%CI 1.187–1.339, *P* < 0.001). Besides, age, gender, primary site, grade, race, TNM stage at diagnosis, surgery and radiotherapy were validated as independent risk or protective factors as well. It is noteworthy that contradictory to common sense, we observed a better 5-year CSS in the no-radiotherapy group (68.3%) than those who received radiotherapy (RT) (58.2%), complicated influence of unadjusted confounders was a possible reason and receiving RT still demonstrated a protective effect on cancer-specific survival (compared with no-RT group, HR: 0.716, 95% CI 0.669–0.767, *P* < 0.001) after multivariate Cox regression (Table 2).

### Impact of marital status on overall survival of OCSCC patients

Subsequently, we also assessed the impact of marital status on OS of OCSCC patients. (Figure 1B) Married group had a better 5-year OS (55.9% vs 41.1%) than unmarried patients and the difference was significant in Log-rank test (*P* < 0.001). Furthermore in the univariate analysis, all the baseline characteristics including gender (*P* = 0.011), age (*P* < 0.001), site (*P* < 0.001), race (*P* < 0.001), year (*P* = 0.002), grade (*P* < 0.001), TNM stage (*P* < 0.001), surgery (*P* < 0.001) and radiotherapy (*P* < 0.001) were also correlated with overall survival and they were further adjusted in multivariate Cox regression (Table 3).

**Table 3:** Univariate and multivariate analysis for evaluating the OS predictors of OCSCC patients

Variable	5-year OS	Univariate Analysis		Multivariate Analysis		
		Log-rank χ^2^	*P*	HR	95%CI	*P*
**Marital status**		329.475	< 0.001			
Married	55.9%			Reference		
Unmarried	41.1%			1.328	1.266–1.392	< 0.001
**Gender**		6.47	0.011			
Female	50.7%			Reference		
Male	48.1%			1.201	1.143–1.262	< 0.001
**Age**		1195.931	< 0.001			
< 35	73.5%			0.136	0.105–0.176	< 0.001
35–44	62.8%			0.232	0.202–0.266	< 0.001
45–54	56.8%			0.281	0.253–0.313	< 0.001
55–64	52.4%			0.355	0.321–0.393	< 0.001
65–74	48.1%			0.457	0.414–0.505	< 0.001
75–84	36.2%			0.694	0.629–0.766	< 0.001
> 85	20.8%			Reference		
**ICD-O-3 site code**		263.598	< 0.001			
Tongue	56.8%			Reference		
Gum and other mouth	44.8%			1.01	0.954–1.070	0.73
Floor of mouth	44.7%			1.231	1.160–1.306	< 0.001
**Race**		130.039	< 0.001			
White	50.5%			Reference		
Black	34.8%			1.191	1.107–1.282	< 0.001
Other	51.2%			0.947	0.866–1.037	0.242
Unknown	77.2%			0.511	0.283–0.924	0.026
**Grade**		180.103	< 0.001			
Well/Moderately differentiated	51.8%			Reference		
Poorly/Undifferentiated	36.8%			1.284	1.212–1.360	< 0.001
Unknown	49.0%			0.963	0.845–1.097	0.568
**Year**		12.226	0.002			
1988–1994	45.9%			Reference		
1995–2001	49.0%			0.906	0.846–0.970	< 0.005
2002–2008	49.9%			0.927	0.867–0.992	0.027
**TNM Stage**		1277.985	< 0.001			
I	68.8%			Reference		
II	52.2%			1.25	1.165–1.341	< 0.001
III	39.8%			1.598	1.470–1.737	< 0.001
IVa/IVb	33.6%			1.88	1.766–2.000	< 0.001
IVc	13.6%			3.101	2.672–3.598	< 0.001
**Surgery**		1609.765	< 0.001			
No surgery	19.0%			Reference		
Local excision/destruction	61.5%			0.512	0.472–0.554	< 0.001
Wide/Radical excision	54.0%			0.528	0.497–0.561	< 0.001
**Radiotherapy**		657.205	< 0.001			
No	59.1%			Reference		
Yes	47.1%			0.787	0.746–0.829	< 0.001

SEER 1988–2008 (*n* = 11022).

In the multivariate analysis, unmarried status significantly increased overall mortality risk (HR 1.260, 95% CI 1.187–1.339, *P* < 0.001). Besides, other covariates including age, gender, primary site, grade, race, stage at diagnosis, surgery, and radiotherapy also proved to be independent prognostic factors for overall survival. The results are summarized in Table 3.

### Effect of marital status on TNM stage at diagnosis

From the baseline characteristics in Table 1, we noticed that married patients had a higher prevalence of stage I/II (57.0%) than unmarried patients (47.5%). We reasonably hypothesized that one important reason for the survival advantage of married group was early diagnosis. If the hypothesis was true, after multivariate adjustment, unmarried individuals should still take higher risk of being diagnosed at a more advanced stage. Relevance between marital status and stage at diagnosis was displayed by univariate and multivariate binomial logistic regression models (Table 4). Race, gender, primary site, grade, age, surgery and radiotherapy were all validated to be related with TNM stage at diagnosis in the univariate logistic regression, and then these variables were adjusted in the multivariate logistic analysis. The result showed unmarried patients were significantly more likely to be diagnosed at stage III or stage IV (compared with married patients, OR: 1.288, 95% CI: 1.182–1.402, *P* < 0.001). Consequently, there was clear evidence that marriage benefited OCSCC prognosis through earlier diagnosis.

**Table 4:** Characteristics of patients by TNM stage at diagnosis using univariate and multivariate binary logistic regression

Characteristics	Stage I/II	Stage III/IV	Univariate Analysis	Multivariate Analysis		
	5792 (100)	5230 (100)	*P*	Odds Ratio	95% CI	*P*
**Marital status**			< 0.001			
Married	3362 (58.0)	2540 (48.6)		Reference		
Unmarried	2430 (42.0)	2690 (51.4)		1.288	1.182–1.402	< 0.001
**Gender**			< 0.001			
Female	2290 (39.5)	1873 (35.8)		Reference		
Male	3502 (60.5)	3357 (64.2)		1.094	1.001–1.196	0.047
**Age**			0.006			
< 35	131 (2.3)	95 (1.8)		0.886	0.629–1.248	0.488
35–44	407 (7.0)	357 (6.8)		1.005	0.786–1.284	0.969
45–54	1104 (19.1)	1060 (20.3)		0.969	0.786–1.195	0.767
55–64	1480 (25.6)	1429 (27.3)		0.93	0.759–1.140	0.485
65–74	1414 (24.4)	1173 (22.4)		0.83	0.678–1.017	0.073
75–84	969 (16.7)	816 (15.6)		0.816	0.663–1.005	0.056
> 85	287 (5.0)	300 (5.7)		Reference		
**ICD-O-3 site code**			< 0.001			
Tongue	2442 (42.2)	1521 (29.1)		Reference		
Gum and other mouth	1763 (30.4)	2083 (39.8)		1.648	1.489–1.824	<0.001
Floor of mouth	1587 (27.4)	1626 (31.1)		1.432	1.288–1.593	<0.001
**Race**			< 0.001			
White	4904 (84.7)	4137 (79.1)		Reference		
Black	398 (6.9))	691 (13.2)		1.521	1.317–1.756	<0.001
Other	464 (8.0)	392 (7.5)		1.094	0.937–1.278	0.256
Unknown	26 (0.4)	10 (0.2)		0.66	0.305–1.426	0.290
**Grade**			< 0.001			
Well/Moderately differentiated	4765 (82.3)	3916 (74.9)		Reference		
Poorly/Undifferentiated	778 (13.4)	1128 (21.6)		1.502	1.346–1.675	<0.001
Unknown	249 (4.3)	186 (3.6)		0.717	0.573–0.896	0.004
**Year**			0.487			NI
1988–1994	788 (13.6)	752 (14.4)				
1995–2001	1680 (29.0)	1492 (28.5)				
2002–2008	3324 (57.4)	2986 (57.1)				
**Surgery**			< 0.001			
No surgery	561 (9.7)	1411 (27.0)		Reference		
Local excision/destruction	1404 (24.2)	549 (10.5)		0.281	0.242–0.327	<0.001
Wide/Radical excision	3827 (66.1)	3270 (62.5)		0.499	0.443–0.562	<0.001
**Radiotherapy**			< 0.001			
No	3856 (66.6)	1634 (31.2)		Reference		
Yes	1936 (33.4)	3596 (68.8)		3.383	3.108–3.681	<0.001

SEER 1988–2008 (*n* = 11022).

Abbreviations: CI: confidence interval; NI: Not Included.

### Relationship between marital status and age at diagnosis

Age was another crucial prognostic factor. To investigate whether married patients were younger at diagnosis than those unmarried, we performed a multivariate multinomial logistic regression to explore the association between marital status and age at diagnosis. When defining the < 35 age subgroup as base outcome for reference, we observed that compared with unmarried patients, married people were more likely to be 55–64 (OR: 1.717, 95% CI: 1.137–2.593, *P* < 0.001), 65–74 (OR: 2.004, 95% CI: 1.508–2.604, *P* < 0.001) and 75–84 (OR: 1.356, 95% CI: 1.015–1.811, *P* = 0.039) years old at diagnosis. However, there was no significant difference between married and unmarried patients in 35–44 (OR: 1.398, 95% CI: 0.667–2.930, *P* = 0.374), 45–54 (OR: 1.670, 95% CI: 0.743–3.755, *P* = 0.215) and > 85 age subgroups (OR: 0.766, 95% CI: 0.552–1.063, *P* = 0.111). Therefore, age might not be the main explanation for survival benefits of marriage (Table 5).

**Table 5:** Multinomial multivariate logistic analysis of age at diagnosis and treatment according to marital status

Variable	Multivariate multinomial logistic analysis		
	Odds Ratio	95% CI	*P*
**Age at diagnosis**			
< 35			
Unmarried	As base outcome		
Married			
35–44			
Unmarried	Reference		
Married	1.398	0.667–2.930	0.374
45–54			
Unmarried	Reference		
Married	1.670	0.743–3.755	0.215
55–64			
Unmarried	Reference		
Married	1.717	1.137–2.593	0.010
65–74			
Unmarried	Reference		
Married	2.004	1.508–2.604	< 0.001
75–84			
Unmarried	Reference		
Married	1.356	1.015–1.811	0.039
> 85			
Unmarried	Reference		
Married	0.766	0.552–1.063	0.111
**Surgery**			
No surgery			
Unmarried	As base outcome		
Married			
Local excision/destruction			
Unmarried	Reference		
Married	1.686	1.459–1.948	< 0.001
Wide/Radical excision			
Unmarried	Reference		
Married	1.606	1.404–1.800	< 0.001
**Radiotherapy**			
No radiotherapy			
Unmarried	As base outcome		
Married			
Receive radiotherapy			
Unmarried	Reference		
Married	1.017	0.932–1.110	0.700

SEER 1988–2008 (*n* = 11022).

### Effect of marital status on treatment selection

Adequate treatment could be another potential cause for longevity among married patients. To investigate whether marital status influenced survival by selection of treatment, we analyzed the preference for receiving surgery or radiotherapy by multivariate logistic regression (Table 5). In the multivariate logistic analysis for surgery, we found that married patients were more likely to undergo surgery, for both local destruction/excision (OR: 1.686, 95% CI: 1.459–1.948, *P* < 0.001) and total/radical surgery (OR: 1.606, 95%CI: 1.404–1.800, *P* < 0.001). Nevertheless in the multivariate logistic analysis for RT, significant preference of radiotherapy was not observed between married and unmarried groups (OR: 1.017, 95% CI: 0.932–1.110, *P* = 0.700).

Therefore, in addition to earlier diagnosis, better prognosis of married OCSCC patients was also attributed to more sufficient treatment, especially surgical treatment.

### Subgroup analysis for evaluating the effect of marital status on OS and CSS

We have certified that delayed diagnosis and undertreatment were important reasons for compromised survival of unmarried patients. Next we separately stratified stage at diagnosis, age and treatment condition, then analyzed survival in each subgroup to see if marriage still benefited patients’ prognosis in subgroup multivariate Cox analysis.

**Figure 2:**
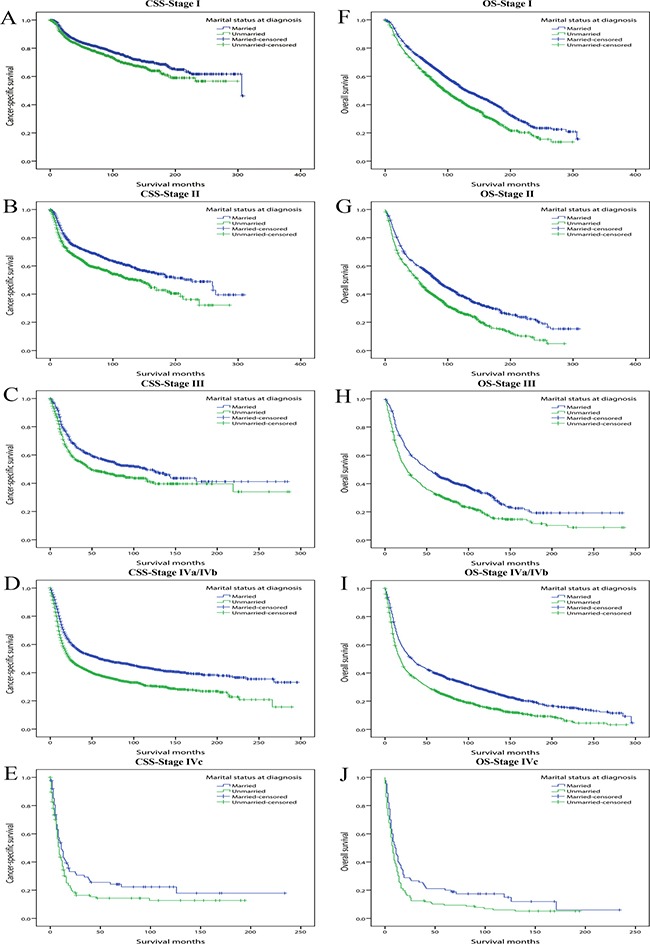
Kaplan-Meier survival curves: cancer-specific survival and overall survival in 11022 OCSCC patients according to TNM stage at diagnosis (**A**) CSS-Stage I: χ^2^ = 8.981, *P* = 0.003; (**B**) CSS-Stage II: χ^2^ =22.635, *P* < 0.001; (**C**) CSS-Stage III: χ^2^ =10.964, *P* < 0.001; (**D**) CSS-Stage IVa/IVb: χ^2^ = 69.246, *P* < 0.001; (**E**) CSS-Stage IVc: χ^2^ =3.581, *P* = 0.058; (**F**) OS-Stage I: χ^2^ =43.523, *P* < 0.001; (**G**) OS-Stage II: χ^2^ =49.323, *P* < 0.001; (**H**) OS-Stage III: χ*2* = 39.349, *P* < 0.001; (**I**) OS-Stage IVa/IVb: χ^2^ =101.648, *P* < 0.001; (**J**) OS-Stage IVc: χ^2^ = 5.398, *P* = 0.020.

Firstly, using multivariate Cox regression, we assessed the effect of marital status on CSS and OS at each stage, in patients with stage I to stage IVa/IVb at diagnosis, marriage always played a significant protective role in both CSS and OS. For overall survival of stage IVc patients, marriage was also a probable beneficial factor though not significant at 95% confidence level (HR: 1.394, 95% CI: 0.989–1.964, *P* = 0.057). Of note, for all the five stage subgroups, the hazard ratios of unmarried patients for OS were always greater than those for CSS, indicating marital status had a stronger impact on overall survival than cancer-specific survival (Table 6) (Figure 2).

**Table 6:** Multivariate subgroup analysis of marital status on OCSCC overall and cancer-specific survival according to TNM stage, age at diagnosis and treatment

Variable	Multivariate Analysis (CSS)			Multivariate Analysis (OS)		
	HR	95% CI	*P*	HR	95% CI	*P*
**TNM Stage**						
Stage I						
Married	Reference			Reference		
Unmarried	1.132	0.984–1.303	0.084	1.216	1.106–1.337	< 0.001
Stage II						
Married	Reference			Reference		
Unmarried	1.339	1.163–1.541	< 0.001	1.37	1.232–1.524	< 0.001
Stage III						
Married	Reference			Reference		
Unmarried	1.205	1.015–1.430	0.033	1.402	1.219–1.613	< 0.001
Stage IVa/IVb						
Married	Reference			Reference		
Unmarried	1.277	1.169–1.396	< 0.001	1.309	1.215–1.411	< 0.001
Stage IVc						
Married	Reference			Reference		
Unmarried	1.246	0.860–1.804	0.245	1.394	0.989–1.964	0.057
**Age at diagnosis**						
< 35						
Married	Reference			Reference		
Unmarried	1.667	0.964–2.881	0.067	1.331	0.800–2.214	0.271
35–44						
Married	Reference			Reference		
Unmarried	1.254	0.972–1.619	0.082	1.459	1.165–1.827	0.001
45–54						
Married	Reference			Reference		
Unmarried	1.335	1.158–1.540	< 0.001	1.487	1.319–1.676	< 0.001
55–64						
Married	Reference			Reference		
Unmarried	1.299	1.155–1.461	< 0.001	1.373	1.250–1.507	< 0.001
65–74						
Married	Reference			Reference		
Unmarried	1.179	1.042–1.336	0.009	1.23	1.120–1.350	< 0.001
75–84						
Married	Reference			Reference		
Unmarried	1.227	1.056–1.424	0.007	1.237	1.108–1.381	< 0.001
> 85						
Married	Reference			Reference		
Unmarried	1.106	0.826–1.480	0.498	1.044	0.849–1.285	0.682
**Treatment**						
No surgery or radiotherapy						
Married	Reference			Reference		
Unmarried	1.328	1.014–1.738	0.039	1.234	0.979–1.555	0.076
Surgery only						
Married	Reference			Reference		
Unmarried	1.251	1.119–1.399	< 0.001	1.331	1.232–1.438	< 0.001
Radiotherapy only						
Married	Reference			Reference		
Unmarried	1.207	1.056–1.381	0.006	1.24	1.108–1.388	< 0.001
Both surgery and radiotherapy						
Married	Reference			Reference		
Unmarried	1.227	1.120–1.344	< 0.001	1.287	1.193–1.384	< 0.001

SEER 1988–2008 (*n* = 11022).

**Figure 3:**
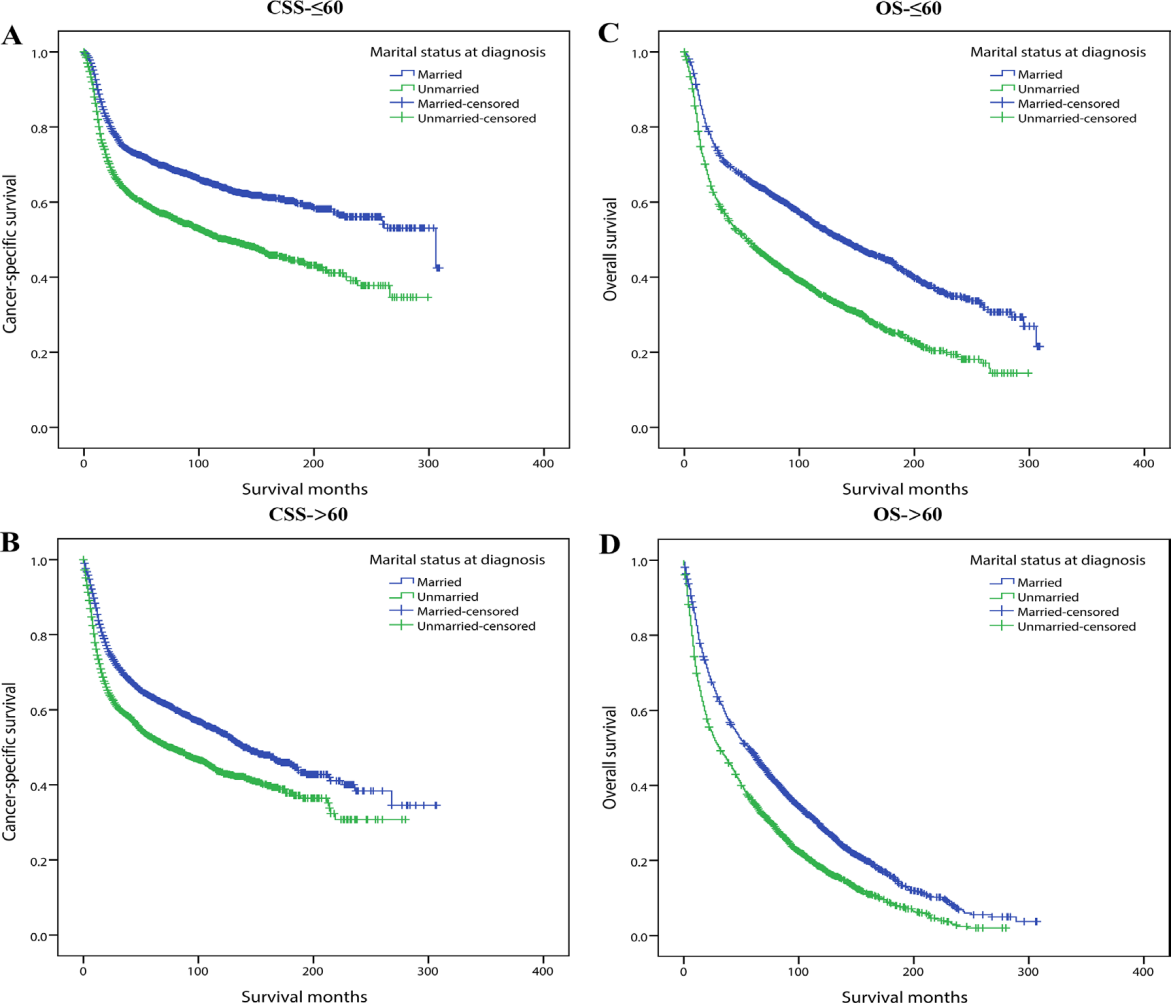
Kaplan-Meier survival curves: cancer-specific survival and overall survival in 11022 OCSCC patients according to ≤ 60 and >60 binary age subgroups (**A**) CSS-≤60: χ^2^ = 104.243, *P* < 0.001; (**B**) CSS->60: χ^2^ = 80.789, *P* < 0.001; (**C**) OS-≤ 60: χ^2^ = 184.233, *P* < 0.001; (**D**) OS-> 60: χ^2^ =146.355, *P* < 0.001.

Secondly, we conducted multivariate Cox regression adjusting for the aforementioned variables and assessed the influence of marital status on survival in each age subgroup. We observed that except for patients younger than 35 or older than 85 years old, unmarried status always significantly increased the mortality risk for both CSS and OS compared with married ones. Similar to stage subgroup analysis, for all the five age subgroups (35–44, 45–54, 55–64, 65–74, 75–84) in which marital status significantly influenced survival, marital status affected OS more greatly than CSS (Table 6). For concise illustration, Kaplan curves for CSS and OS were conducted based on binary age (≤ 60 and > 60) subgroups (Figure 3).

**Figure 4:**
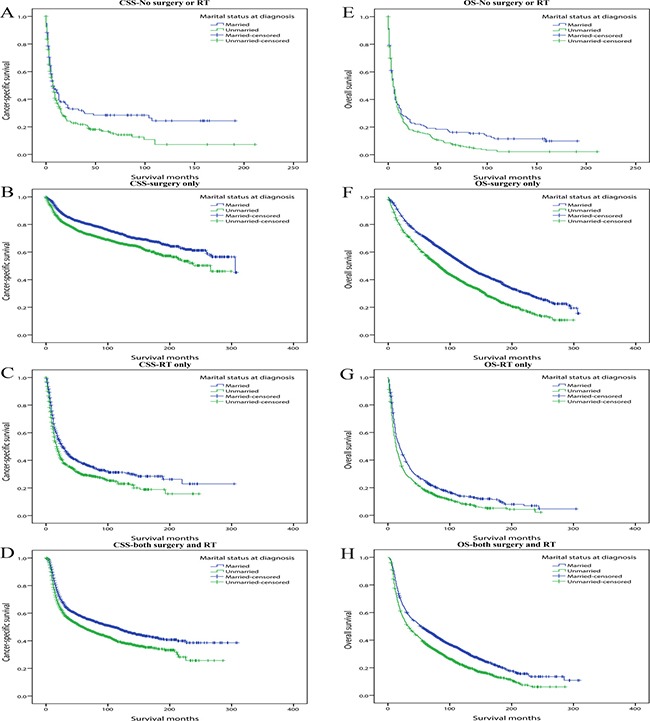
Kaplan-Meier survival curves: cancer-specific survival and overall survival in 11022 OCSCC patients according to treatment (**A**) CSS-No surgery or RT: χ^2^ = 5.400, *P* = 0.020; (**B**) CSS-Surgery only: χ^2^ =35.985, *P* < 0.001; (**C**) CSS-RT only: χ^2^ =14.550, *P* < 0.001; (**D**) CSS-Both surgery and RT: χ^2^ =31.287, *P* < 0.001. (**E**) OS-No surgery or RT: χ^2^ = 4.389, *P* = 0.036; (**F**) OS-Surgery only: χ^2^ = 116.459, *P* < 0.001; (**G**) OS-RT only: χ^2^ = 19.604, *P* < 0.001; (**H**) OS-Both surgery and RT: χ^2^ = 62.052, *P* < 0.001.

Thirdly we divided patients into four subgroups which were No surgery or radiotherapy, Surgery only, Radiotherapy only and Both surgery and radiotherapy subgroups. In the multivariate Cox analysis, marriage always demonstrated protective role and marital status demonstrated stronger influence on OS than CSS (Table 6) (Figure 4).

**Figure 5:**
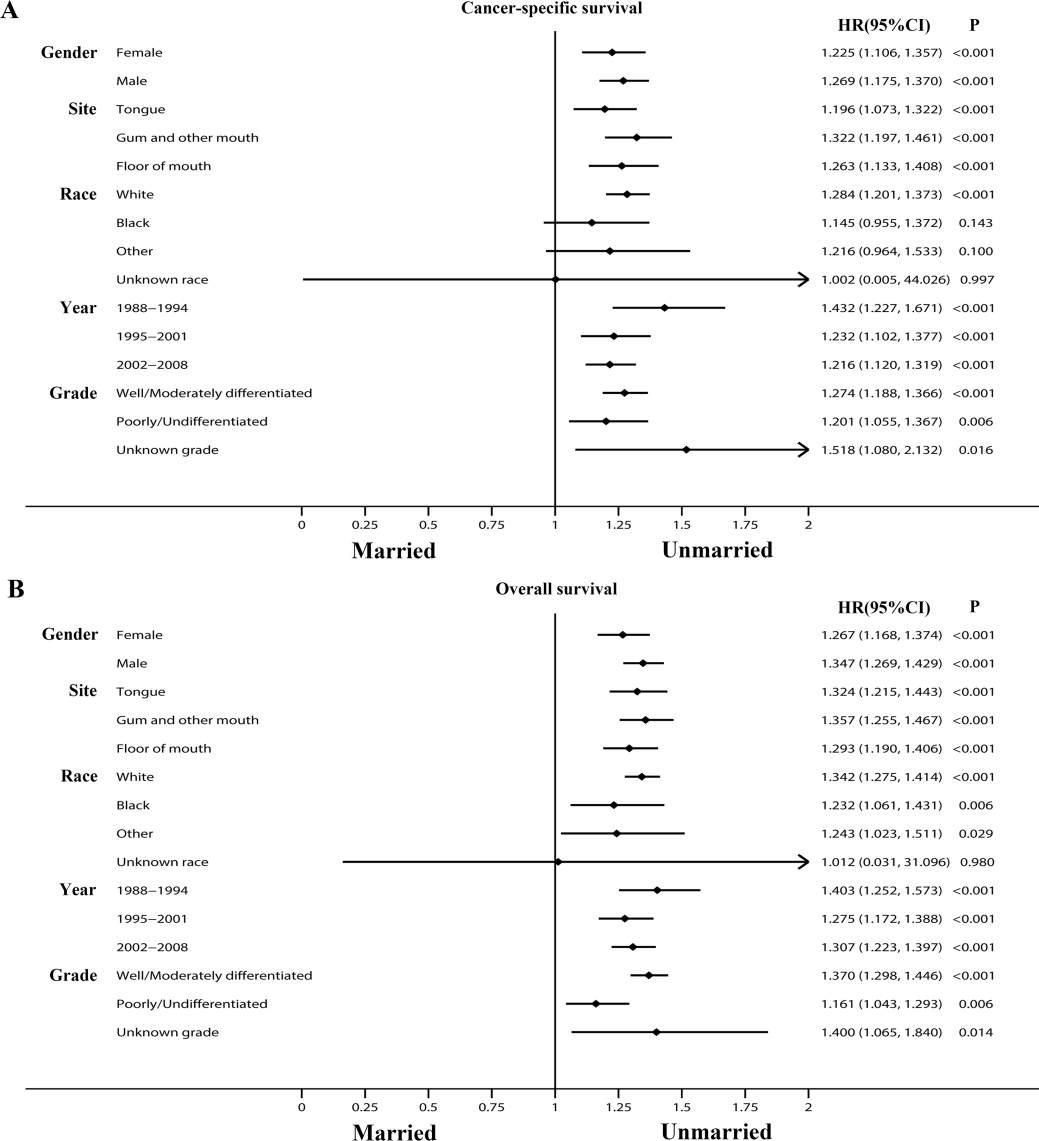
Forest plots summarizing hazard ratios for cancer-specific survival and overall survival in subgroup analyses: Married versus Unmarried The X-axis displays the hazard ratio and 95% CI of each subgroup, ticks are arranged at 0, 0.25, 0.5, 0.75, 1.0, 1.25, 1.5, 1.75, and 2.0. (**A**) Cancer-specific survival. (**B**) Overall survival.

In addition, we conducted subgroup analyses stratified by other baseline characteristics including gender, grade, primary site, year of diagnosis and race. The results are briefly summarized in two forest plots (CSS: Figure 5A; OS: Figure 5B). It was found that for both CSS and OS, compared with female, male patients displayed higher mortality risk when they were unmarried, illustrating marriage and its related spousal support might be more effective in men than women (CSS: HR for male unmarried group: 1.269, HR for female unmarried group: 1.225; OS: HR for male unmarried group: 1.347, HR for female unmarried group: 1.267). The impact of marital status was also stronger in white population than other ethnicity, gum squamous cell carcinoma patients than any other primary site, well/moderately differentiated tumor grade than those poorly differentiated or anaplastic.

### Survival analysis in 1:1 matched group

As we’ve discussed above, married patients favored earlier diagnosis and more adequate treatment thus to benefit survival. Nevertheless, besides of these reasons, spousal support may also improve OCSCC prognosis from other aspects. In order to minimize the disturbance of confounding factors and ensure these confounders were not responsible for the outcomes, we utilized propensity score matching (PSM) method to perform a 1:1 matched cohort. Particularly, exact matching function was used for age, grade, TNM stage, surgery and radiation to make sure married and unmarried groups were identical in these variables. After matching, we obtained 6208 patients including 3104 married and another 3104 unmarried ones. Demographics and clinicopathological characteristics of the matched cohort are presented in Table 7. Besides of the exactly matched covariates which were totally the same (*P* = 1), other factors including gender (*P* = 0.854), site (*P* = 0.976), year (*P* = 0.295), race (*P* = 0.893) showed no significant difference as well.

**Table 7:** Characteristics of patients by marital status in 1:1 matched group

Characteristic	Total	Married	Unmarried	*P*
	6208 (100)	3104 (100)	3104 (100)	
**Gender**				0.854
Female	2297 (37.0)	1152 (37.1)	1145 (36.9)	
Male	3911 (63.0)	1952 (62.9)	1959 (63.1)	
**Age**				1
< 35	108 (1.7)	54 (1.7)	54 (1.7)	
35–44	370 (6.0)	185 (6.0)	185 (6.0)	
45–54	1234 (19.9)	617 (19.9)	617 (19.9)	
55–64	1790 (28.8)	895 (28.8)	895 (28.8)	
65–74	1532 (24.7)	766 (24.7)	766 (24.7)	
75–84	980 (15.8)	490 (15.8)	490 (15.8)	
> 85	194 (3.1)	97 (3.1)	97 (3.1)	
**ICD-O-3 site code**				0.976
Tongue	2188 (35.2)	1090 (35.1)	1098 (35.4)	
Gum and other mouth	2176 (35.1)	1091 (35.1)	1085 (34.9)	
Floor of mouth	1844 (29.7)	923 (29.7)	921 (29.7)	
**Race**				0.893
White	5451 (87.8)	2735 (88.1)	2716 (87.5)	
Black	362 (5.8)	178 (5.7)	184 (5.9)	
Other	377 (6.1)	182 (5.9)	195 (6.3)	
Unknown	18 (0.3)	9 (0.3)	9 (0.3)	
**Grade**				1
Well/Moderately differentiated	5230 (84.2)	2615 (84.2)	2615 (84.2)	
Poorly/Undifferentiated	872 (14.1)	436 (14.1)	436 (14.1)	
Unknown	106 (1.7)	53 (1.7)	53 (1.7)	
**Year**				0.295
1988–1994	751 (12.1)	381 (12.3)	370 (11.9)	
1995–2001	1557 (25.1)	802 (25.8)	755 (24.3)	
2002–2008	3900 (62.8)	1921 (61.9)	1979 (63.8)	
**TNM Stage**				1
I	2076 (33.4)	1038 (33.4)	1038 (33.4)	
II	1206 (19.4)	603 (19.4)	603 (19.4)	
III	622 (10.0)	311 (10.0)	311 (10.0)	
IVa/IVb	2250 (36.2)	1125 (36.2)	1125 (36.2)	
IVc	54 (0.9)	27 (0.9)	27 (0.9)	
**Surgery**				1
No surgery	890 (14.3)	445 (14.3)	445 (14.3)	
Local excision/destruction	900 (14.5)	450 (14.5)	450 (14.5)	
Wide/Radical excision	4418 (71.2)	2209 (71.2)	2209 (71.2)	
**Radiotherapy**				1
No	3042 (49.0)	1521 (49.0)	1521 (49.0)	
Yes	3166 (51.0)	1583 (51.0)	1583 (51.0)	

SEER 1988–2008 (*n* = 6208).

*P* value was analyzed by Pearson χ^2^ test.

**Figure 6:**
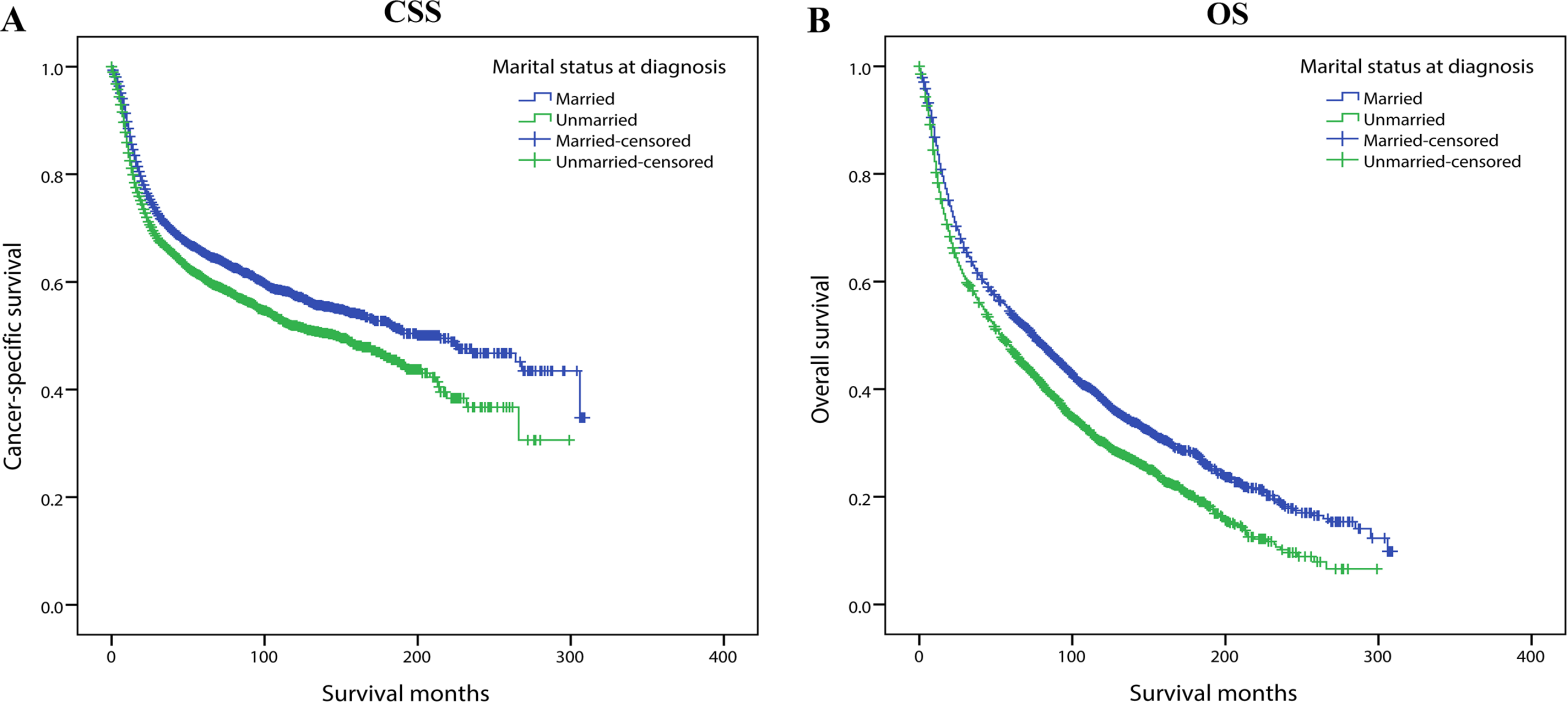
Kaplan-Meier survival curves of 1:1 matched group: cancer-specific survival and overall survival in 6208 OCSCC patients Married vs Unmarried (**A**) Cancer-specific survival: χ^2^ = 21.669, *P* < 0.001. (**B**) Overall survival: χ^2^ = 49.785, *P* < 0.001.

Even so, in the survival analysis, married patients still demonstrated better prognosis than unmarried ones. The 5-year CSS rate of married patients was 65.4% while the proportion of 5-year CSS was 60.6% in unmarried group. Similarly, the 5-year OS rate was 54.1% in the married group and 47.4% in unmarried group (Figure 6). In order to enhance persuasion, we also conducted multivariate Cox regression although all the confounding variables were basically the same between two groups. Unmarried status was still an independent risk factor for both CSS (HR: 1.231, 95% CI: 1.137–1.332, *P* < 0.001) and OS (HR: 1.279, 95% CI: 1.203–1.360, *P* < 0.001). These results suggested that marital status-related spousal support was likely to improve outcomes beyond levels of diagnosis and treatment. Details on it are discussed further in the following section.

## DISCUSSION

It has been confirmed that married patients possessed lower mortality rate and favored a longer life expectancy in different cancer types [8–11]. However, impact of marital status on OCSCC survival has not been fully discovered, and few of these previous studies have focused on the underlying mechanisms of the survival advantage associated with marriage. Additionally, previous research focusing on the relationship between marital status and head neck cancer outcome ignored overall survival and concentrated merely on cancer-specific survival [12, 13]. For the first time, our study indicated that marriage had an independent beneficial influence on not only cancer-specific survival and but overall survival of OCSCC patients as well. Particularly, our research indicated that marital status could independently predict survival in most of the stage, age, treatment, gender and race subgroups and exert a greater effect on overall survival than cancer-specific survival. Furthermore, it is the first study to clarify the reasons why marriage protects OCSCC patients from mortality in depth.

It has been shown that marriage-related spousal support improved prognosis of cancer patients in many ways. Prior literatures confirmed married people tended to undergo earlier screening and obtained more adequate treatment [7, 10]. Our study also reached similar conclusions. As can be seen in our results, multiple reasons such as earlier stage at diagnosis, preference of receiving surgery contributed to the survival advantage of married OCSCC individuals. Though widowed patients in the unmarried group was generally of much higher age and showed worse prognosis in other cancer types [8, 9], it should be noted that in our study, elder age might not account for poorer prognosis of unmarried patients. When we combined those widowed with divorced and never married population as a whole unmarried category then carried out multivariate logistic analysis, age of unmarried group was no longer higher than those married.

Moreover, independent of tumor characteristics and treatment status, the persisting survival advantage of married patients in the 1:1 matching analysis could be hypothetically explained by socioeconomic and psychological factors. The importance of quality of life (QOL), which included both psychological and sociocultural indicators, was emphasized in head and neck cancer survival by Ringash et al, although the authors didn't link QOL with marital status directly [14]. It has been proposed that psychology, living habits, economic conditions, as well as certain biological factors were all strongly associated with marriage. As is well-known, smoking and alcoholism have proved to be intimately correlated to etiology and prognosis of OCSCC [2, 15]. Lindström and Stack et al. separately reported that marriage led to less nicotine intake and alcohol consumption [16, 17]. Hence, marriage was likely to help OCSCC patients give up such bad behaviors to reduce the additional harm [18]. Male and black population possessed higher rates of smoking and drinking [19, 20], consequently it could explain why marriage had a stronger effect on survival of men and black people in our study. Psychologically, married patients had easier access to solicitude or assistance from their family and friends, and possessed higher levels of fighting spirit and lower levels of distress [21, 22]. Brown et al. reported depression symptomology was the most consistent predictor of shorten survival time in cancer patients [23]. Another research by Van der Meulen et al. also proved that head and neck cancer including OCC patients benefited from psychological interventions [24]. Therefore, more attention should be paid to psychological problems of unmarried OCSCC patients. Additionally, married people tended to have better economic status and were more likely to be insured, which can provide them better treatment and nursing conditions [25, 26]. Physiologically, cortisol level and immunity indicators like NK cell activity, T-cell infiltration ability were all associated with distress and depression. They could be improved by spousal support more or less [27–30].

Not only cancer, marriage also played a positive role in overall health. Kubzansky reported marriage protected people from type 2 diabetes through favorable changes in lifestyle [31]. Another study of African Americans with heart failure proved that being married and living with family independently predict lower mortality and fewer readmissions [32]. Besides, a research of East Asian populations revealed marriage and marital satisfaction was of great importance in determining self-rated health [33]. These studies were compatible with our results that the greater impact of marital status on OS than CSS.

The findings of our study could help clinicians understand more about the role of marital status in prognosis of oral cavity cancer, thus allow them to pay more attention to social support of unmarried patients. For example, when a man demonstrating a higher risk of oral cavity cancer came to hospital, we could leave his contact information. If he is married, we can tell his spouse to focus on oral cavity condition of the patient and remind him not to neglect his potential illness. In other words, the spouse could take on partial responsibility of social support, and thereby reduces the burden on the social health network and family physicians. However, if the patient is unmarried, he lacks the spousal support which can offer health recommendations. Utilizing the community health network or care of the family doctor, compared to a married patient, we need to remind him more frequently to pay attention to the condition of oral cavity and take examinations routinely. By providing social support in this way, we can replace the role of the spouse to some extent.

Inevitably, potential limitations of our research should be taken into consideration. Firstly, in SEER database, information of some important therapies was not accessible, such as chemotherapy and biotherapy. Secondly, smoking, alcohol and HPV status were important risk and potential prognostic factors for OCSCC, marriage may also perform its protective role through less nicotine and alcohol intake, just as we’ve discussed above. However, these factors were not recorded in SEER database either. This limitation might lead to potential bias. Thirdly, duration of marriage could possibly affect the effect of marital status as well. But SEER database only collected marital status at the time of diagnosis, numerous patients enrolled in our study survived a long time, during the follow-up period, marital status might change and affect survival. Besides, marital satisfaction records were lacking, which was a crucial component of marital quality. Fourthly, some important etiologic factors were not recorded in SEER like tobacco and alcohol use. Finally, factors like education and income status were also likely to play a role. Ansell et al. and Boyd et al. separately proved that higher income was associated with lower cancer incidence and better prognosis, more exposure to carcinogens and lower rate of insurance were considered to be possible explanations [34–35]. Two studies based on large databases in Sweden and Finland reported that higher education level helped to achieve higher socioeconomic status, which was often related to earlier detection and adequate treatment, thus to improve prognosis [36–37]. Lack of educational and income status in SEER database might increase the possibilities of bias.

Despite these limitations above, our study confirmed the survival disparity of OCSCC patients related with marital status. In summary, married patients always showed a significant survival advantage than those unmarried. The results were derived from timely diagnosis, adequate treatment and probably influenced by spousal support through psychological, economical and physiological ways. Consequently, our findings heighten the awareness of the effect of marital status on OCSCC outcome and encourage clinicians to provide unmarried oral cavity cancer patients and high-risk groups with more social support, in order to help them get a better outcome, for both longer survival time and higher life quality.

## MATERIALS AND METHODS

### Data source

We obtained data from Surveillance, Epidemiology, and End Results (SEER) database sponsored by National Cancer Institute. SEER database collects information of cancer patients in 18 registries, covering about 28 percent of total U.S population. The database includes some important oncological data, such as demographics, primary sites, morphology, stage, surgery, radiotherapy, grade and patients’ vital status. SEER*Stat 8.3.2 software was used to extract information from the database.

### Inclusion criteria

The inclusion criteria were as follows: 1) Known marital status. 2) 18 years old or older at the time of diagnosis. 3) Diagnosed with OCSCC only or multiple primary cancers but OCSCC was the first. 4) The ICD-O-3 site codes were limited to C02.0–02.3, 02.8–02.9, 03.0–03.9, 04.0–05.0, 05.8–06.9. It's worth noting that codes 00.0–00.9 (Lip) were not included because NCCN regarded lip and oral cavity as two different parts and provided different treatment guidelines respectively [38]. Subsites of oropharynx were also excluded like base of tongue, tonsil, soft palate and uvula, etc. 5) Histological type was limited to squamous cell carcinoma (8052, 8070–8078, 8083–8084) according to ICD-O-3 histological codes. 6) Diagnosed between 1/1/1988 and 12/31/2008 to ensure an adequate 5-year follow-up time, as the follow-up cutoff date of currently available SEER data was 12/31/2013. 7) Active follow-up. 8) Known survival months after diagnosis and known cause of death. 9) Known surgery condition and radiotherapy condition 10) Definite AJCC TNM stage at diagnosis.

### Study variables

Patient's important characteristics were extracted from SEER database to be our study variables, including marital status, TNM stage at diagnosis, age at diagnosis, gender, primary tumor site, tumor grade, race, surgery condition and radiotherapy condition. AJCC TNM stage IV was divided into stage IVa/IVb and stage IVc since stage IVc meant distant metastasis and its prognosis could be rather different from stage IVa and IVb. Marital status which was the primary variable of interest was analyzed as a bivariate value (married and unmarried).

### Statistical analysis

Baseline characteristics were compared by χ^2^ test. The primary end points of our study were CSS and OS, which were analyzed by Kaplan-Meier analysis and the difference was calculated by Log-rank test. Relationship between marital status and TNM stage at diagnosis, age at diagnosis and treatment were conducted by binomial or multinomial logistic regression. Multivariate Cox proportional hazards regression models were built for presenting hazard ratios of different study variables. We also used forest plots to summarize risk factors for survival in subgroup analyses. A 1:1 paired cohort matching with marital status was performed by propensity score matching (PSM) method.

Kaplan-Meier curve, logistic regression, multivariate Cox regression and χ^2^ test were analyzed by statistical software IBM SPSS, version 22 (SPSS Inc, Chicago, IL, USA). Propensity score matching method and forest plots were conducted by Stata statistical software, version 14.0 (StataCorp, College Station, TX). All *P* values were two-sided and statistical significance was set at *P* < 0.05.
